# Electromigration in Nano-Interconnects: Determining Reliability Margins in Redundant Mesh Networks Using a Scalable Physical–Statistical Hybrid Paradigm

**DOI:** 10.3390/mi15080956

**Published:** 2024-07-26

**Authors:** Houman Zahedmanesh

**Affiliations:** IMEC, Kapeldreef 75, B-3001 Leuven, Belgium; houman.zahedmanesh@imec.be

**Keywords:** nano-interconnects, reliability, electromigration (EM), redundancy, mesh networks, power delivery network, physics-based modelling, statistical electromigration budgeting (SEB), PDN unit-cell-based/tile-based SEB

## Abstract

This paper presents a hybrid modelling approach that combines physics-based electromigration modelling (PEM) and statistical methods to evaluate the electromigration (EM) limits of nano-interconnects in mesh networks. The approach, which is also compatible with standard Place and Route (P&R) tools and practises, takes into account the positive impact of network redundancy on EM current limits. The numerical simulations conducted in this study show that conventional methods underestimate the EM current limits of a power delivery network (PDN) unit-cell by 80% due to their lack of consideration for redundancy. Additionally, the time-to-failure (TTF) distributions of a PDN unit-cell obtained by the developed modelling framework adhered to a lognormal distribution, where the lognormal sigma, σ_logn_, exhibits a 55% reduction compared to that of the single constituent interconnects. The study also found the negative voltage (i.e., ground or V_ss_) grid to be more susceptible to EM than the positive voltage, i.e., V_dd_ grid. In the examined grid unit-cell design, both the number of interconnect sites prone to voiding and also the magnitude of the peak tensile stress within the nano-interconnects were found to be two times as high in the V_ss_ case compared to V_dd_. The lognormal sigma of TFF for the grid unit-cells, $\sigma_{logn-tile}$, show a marked reduction compared to the lognormal sigma of the constituent single interconnects, $\sigma_{logn}$, with a 50% and 66% decrease compared to single interconnects, for downstream (V_ss_) and upstream (V_dd_), respectively. In addition, $\sigma_{logn-tile}$ was three times higher for downstream (V_ss_) compared to upstream (V_dd_), whilst, in contrast, this difference was only 2-fold at the single interconnect level. TTF_50%_ was predicted to be 4.13-fold higher at the grid unit-cell level for the upstream compared to downstream operation, which was also more pronounced than in the single interconnect level where the difference was only 2-fold. This research provides valuable insights into the EM ageing of nano-interconnects in mesh networks and could pragmatically enhance the accuracy of EM compliance evaluation methods.

## 1. Introduction

The rapid integration of electronics into daily applications, such as the automotive industry and the Internet of Things (IoT), underscores the need for research and innovation in design-for-reliability paradigms more than ever. In the realm of Very Large Scale Integration (VLSI) reliability, the use of overly conservative approaches, which involve large reliability margins and safety factors, restricts the design space, thereby affecting chip performance and power consumption. Given the anticipated computing energy demands associated with the rise and prevalence of artificial intelligence (AI) [1], along with the complex consequences of materials and manufacturing processes, these overly pessimistic reliability paradigms are at odds with global sustainability requirements and the sustainability obligations of the semiconductor sector. 

One of the key (VLSI) reliability challenges concerning metal interconnections is electromigration (EM). EM is a phenomenon where metal atoms are displaced due to the momentum transfer from conducting electrons [2,3]. This displacement can lead to the formation of voids in interconnects, causing circuit failures. The failure occurs by void nucleation and growth at the cathode end of interconnects, which results in an increase in interconnect resistance, impairing circuit operation. For instance, it can induce timing errors and eventually lead to open circuit failures. The impact of EM on the reliability of VLSI circuits has been a topic of extensive research as a central concern of interconnect reliability [4,5]. 

In standard EM tests, interconnects are characterized by determining their time-to-failure (TTF) under constant direct current (DC) tests with flux divergence points at their two ends. An interconnect is considered to have failed when its resistance increases beyond a target value, often a 10% resistance shift (R Shift). Tests are conducted at accelerated conditions by increasing the current density and temperatures. Multiple interconnects are tested under different temperatures and current conditions and the mean time-to-failures (MTTF) at different conditions are used to determine the activation energy, E_a_, and the current density exponent, n, of Black’s equation [2,6,7,8]. TTFs follow a lognormal distribution, whereas the lognormal and Black’s equation parameters are used to derive an EM current density limit, j_max_, for a target EM failure probability and lifetime [9,10]. The current density limit, j_max_, is usually reported in the process design kits (PDKs) provided by the foundry to be employed by the designer for EM compliance checks in the Place and Route (P&R) phase.

Fundamentally speaking, EM is correlated with the cohesive energy of metals and therefore also correlated with their meting point. Many widely used metals in CMOS technology such as Al and Cu suffer from EM. In contrast, for metals with a high melting temperature such as Ru, Mo and W, EM is not a major reliability concern [11,12]. Many material and process-related factors are implicated in EM, including interfacial properties, e.g., adhesion [13], Cu microstructure (i.e., grain size distribution) [14,15,16,17,18,19], mechanical and fracture properties of the metal, confining materials and dielectrics and residual stresses [20,21,22,23,24,25,26]. Thereby, technological solutions involve material and process innovations, such as using dopants (e.g., AL and Mn) that segregate into Cu grain boundaries, and also the implementation of liners and metal capping, e.g., Co cap [27,28,29], each with their pros and cons in terms of resistivity and cost [30,31]. 

With an increase in current densities and the drastic decline of EM robustness due to the miniaturization of interconnects [16,32,33,34], EM is considered to be a significant reliability challenge for ongoing scaling [35]. As scaling progresses, delay shifts attributed to EM are anticipated to overshadow other ageing mechanisms such as hot carrier injection (HCI) and bias temperature instability (BTI) [36]. Despite the advent of EM-robust alternative metals such as Mo, and Ru projected for use in angstrom nodes [11,12], resistivity considerations restrict their application to wires narrower than ~12 nm linewidths [37]. Thus, the back-end-of-line (BEOL) will continue to display a hierarchical architecture where wider metal levels will be Cu-based given its cost and resistivity benefits [37]. Moreover, the rise of back-side power delivery networks (BS-PDNs), where the PDN is processed on the opposite side of the wafer [38,39], justifies the use of Cu in back-side power delivery applications given available spacing for more relaxed linewidths. In this scenario, transistor heat generation and interconnect joule heating in the absence of effective chip cooling options [40] will raise Cu interconnect temperatures and temperature gradients, thereby intensifying EM ageing [41,42]. Evidently, EM will persist as a reliability concern as we venture into the angstrom technology era.

The standard design-for-reliability approaches widely employed for EM by the industry are as follows: (i) the limit-based approach (LBA), which predicts system failure when the average DC current density, j, exceeds j_max_, for any interconnect, ignoring the statistical nature of EM [10,43] and (ii) statistical EM budgeting (SEB), which employs the weakest link statistics considering the distribution of the j/j_max_ ratio of all single interconnects [10,43]. Thereby, in these methods, failure is predicted when j_max_ is violated in any single interconnect or the failure probability is predicted based on a first-to-fail interconnect criterion, respectively. This assumption is plausible for circuits where interconnects are connected in series. However, the PDN, which is most prone to EM because of the high average magnitudes of unipolar currents, has a grid-like architecture with many parallel interconnect paths. Thereby, if one interconnect suffers from EM, the current will be redirected to redundant parallel paths. Clearly, application of the conventional EM compliance evaluation methods to PDN may be overly pessimistic, as the formation of the first void alone does not necessarily cause a system failure [44].

To this end, many studies have been dedicated to the investigation of EM in grid-like networks using both experimental and simulative approaches. Zhou et al. (2018) used a test chip to study EM effects in PDNs, and observed mechanical stress-dependent failure locations in grids and self-healing due to redundant current paths [45]. Using a similar on-chip approach, Pande et al. (2019) captured several EM effects including abrupt and/or progressive failures, temporary healing effects and circuit–interconnect interplay, which may not be observed through single interconnect characterization [46]. Lin et al. conducted systematic experiments on the impact of redundancy by using test structures with a different number of parallel interconnects, and proposed a statistical model to predict the TTF of parallel interconnect networks based on the TTF of the last failing interconnect [47]. As the latter model was purely statistical, the physical dynamics, e.g., the current redirection and accelerated ageing of the late-failing interconnects, could not be captured. Yet the approach explained many of the key EM characteristic trends of parallel systems such as the decrease in the lognormal sigma, $\sigma_{logn}$, with an increase in redundancy [47]. With the increasing complexity of grid architecture, however, their model predictions diverged from experimental findings, possibly due to neglecting the time-dependent physical cascade of phenomena such as current and stress redistribution within the grids. In this context, an understanding of the distribution of EM-induced hydrostatic stress within the grid has been shown to be the pre-requisite to determine the EM failure locations [48]. EM voids occur in locations of high tensile stress which do not strictly coincide with locations of peak current within a grid and require grid-level stress analysis [48]. To this end, physics-based modelling of EM has seen significant advances in recent years considering the transient simulation of all stages of EM ageing where models have become more technology and microstructure aware [32,49,50,51,52,53,54,55,56,57,58,59]. However, the application of such exhaustive models to grids with billions of segments entails significant computational costs. This is mainly because the simulation of the post-voiding cascade of events requires transient coupled electrical-EM analysis to capture current redistribution within the grid. To minimize such computational costs, model order reduction together with filtering algorithms that confine the analysis to critical interconnect segments have been adopted in the literature [60,61,62,63]. Furthermore, due to their computational expense, complexity, and parametric uncertainty, the statistical aspect of EM is frequently overlooked. This often results in models failing to deliver the crucial chip-level failure probability. In addition to the need for electronic design automation (EDA) software packages capable of resolving the transient mechanical stress distribution across the entire chip before and after void-nucleation, the adoption of stress-based approaches necessitates the provision of mechanical stress limits (i.e., critical stress) from the foundry. These limits can only be indirectly inferred, for instance, through model-based approaches. Therefore, despite their inherent limitations, current-based EM compliance evaluation methods continue to be the industrial benchmark.

**Figure 1:**
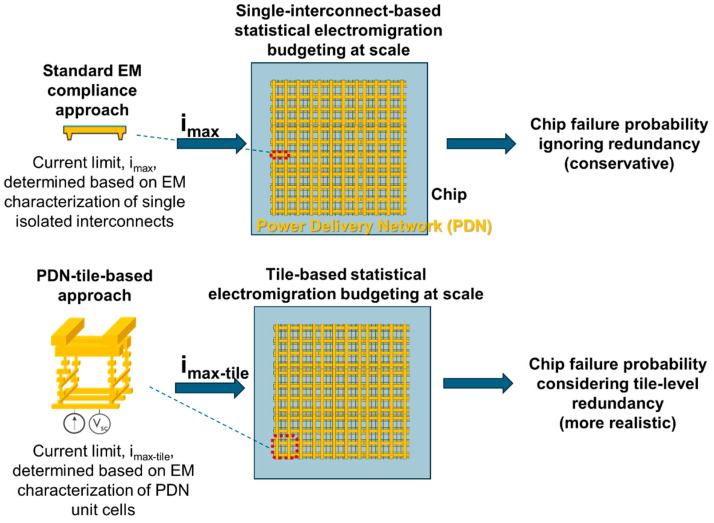
The PDN-tile-based EM compliance evaluation approach proposed in [64] as compared to the standard approach.

To overcome the described practical constraints and account for grid redundancy in chip-level reliability predictions, we recently introduced the concept of a PDN-tile-based EM compliance check; see Figure 1. This method derives current limits for the unit-cells (or tiles) of the PDN. This approach is practical because PDNs are architecturally composed of repeating grid unit-cells, each consisting of parallel interconnect paths. Consequently, the redundancy impact is inherently captured in the current limits determined at the tile-level characterization. Further, tile-based SEB is be applied, where PDN tiles are considered as the fundamental elements for the weakest link statistics, instead of single interconnects; see Figure 1. This allows for a scalable EM assessment of PDNs, considering the impact of PDN redundancy [64]. 

## 2. Key Contributions and Outline

This paper presents a predictive numerical modelling framework and specifically an efficient method for simulating variability propagation from the single interconnect level to PDN unit-cell level. This development further consolidates the PDN unit-cell-based EM compliance checks that we proposed in [64]. However, in [64], the TTF variability was only attributed to the variability in time-to-nucleation by considering the critical stress as the main variable. In this study, the variabilities and their propagation from the single interconnect level to PDN unit-cell level are treated more rigorously. Variabilities in both nucleation and void growth phases are considered using an efficient order-reduced model that can also capture the impact of void dynamics. As such, the variabilities stemming from void shape and location which are postulated to be the main cause of differences in upstream and downstream EM can be captured. The latter enabled the model to decipher fundamental differences between EM in the negative supply voltage grid (V_ss_ grid), and the positive supply voltage grid (V_dd_ grid), at the PDN unit-cell level. This is an advancement with respect to the existing probabilistic models of EM in PDN such as in the study by Mishra and Sapatnekar (2017) [65]. The modelling framework is used to provide quantitative predictions of TTF distributions for a PDN unit-cell. This unit-cell features a complex multi-layer architecture, making it relevant for advanced technology nodes. The model offers invaluable insights into the quantitative impact of grid redundancy and operation mode (i.e., V_dd_ vs. V_ss_ grid). Furthermore, it contextualizes the reliability metrics derived from the PDN unit-cell by comparing them with metrics derived from the single interconnect level. 

The structure of the paper is summarized as follows:

Section 3.1: A description of the developed physics-based numerical modelling framework that is devised to model the ageing of PDN unit-cells is provided. The framework is developed by coupling an EM physics-based modelling module with a circuit solver module.

Section 3.2: The EM modelling module is described. This module simulates EM in every interconnect of the PDN, within each simulation time step.

Section 3.3: An efficient approach to model EM variability propagation from the single interconnect level is provided. This enables the prediction of reliability metrics for complex PDN unit-cells based on metrics obtained from standard single interconnects.

Section 4.1: The modelling framework is corroborated by comparing the model predictions with experimental findings for a simple double redundancy interconnect system consisting of two parallel interconnects working in concert.

Section 4.2: The modelling framework is used to predict the TTF distributions for a PDN unit-cell with a highly complex multi-layer architecture relevant for advanced nodes.

Section 4.3: A running example is provided which quantitatively demonstrates the impact of considering redundancy in a PDN unit-cell on reliability metrics such as maximum permissible standard cell currents.

## 3. Method and Materials

### 3.1. Network-Aware Modelling Framework 

A network-aware EM modelling framework was devised and developed by coupling a circuit solver module with a Korhonen-type [49] EM modelling module described in Section 3.2. The Korhonen model has the advantage of being a simple 1D physics-based model that can efficiently resolve the EM-induced mechanical stress distribution along interconnects. Thereby, it can accurately predict void nucleation based on critical stress criteria. The electrical current in each interconnect segment of the network was derived using the circuit solver and communicated to a 1D partial differential equation solver which solved the Korhonen EM model over each interconnect to determine the evolution and distribution of hydrostatic stress along its segments. Once a critical tensile stress was reached at a location along the interconnects, void nucleation was considered and a compact resistance evolution model determined the increase in the interconnect segment’s electrical resistance. The resistance increase was considered in the subsequent simulation time step to determine current redistribution through the network due to EM voids. In terms of numerical implementation, the electrical circuit solver module and the EM solver module were coupled by sharing log files in every time step of an explicit solution approach. The minimum stable time step was determined through sensitivity analyses. The simulation framework was implemented within MATLAB^®^ 2022a environment using Simscape in Simulink for electrical circuit analyses and the PDEPE partial differential equation solver for the solution of the Korhonen-type EM model, where the communication and coupling of the modules were conducted using custom-developed programmes. 

**Figure 2:**
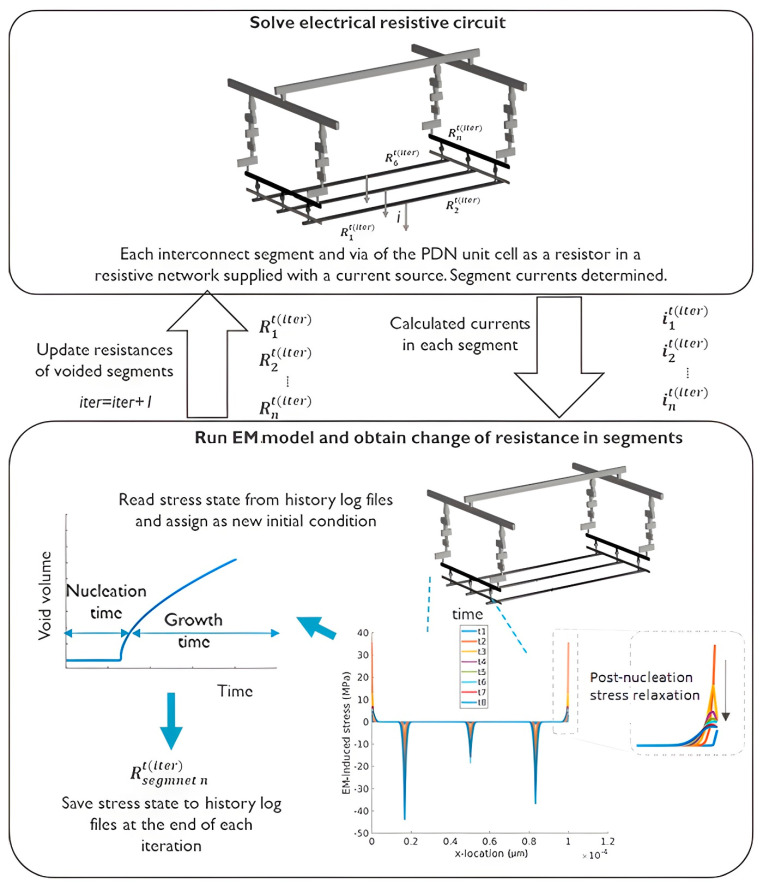
The diagram of the electrical-EM coupling approach in the simulation framework.

The electrical circuit was modelled as a resistive network, where the resistance of each line segment, each via and their connectivity were pre-assigned per interconnect design and technology assumptions. The operation of the circuit was simulated with ideal current sources representing the standard cells or stress current injectors, where the circuit operated under upstream (US) or downstream (DS) conditions by changing the direction of the ideal current sources. The algorithmic diagram is demonstrated in Figure 2.

### 3.2. Electromigration Model

During EM, the electron wind force depletes the ions in the direction of the electron flow while a back stress develops and drives ions in the opposite direction, so that [49]

(1) $$J=-\frac{DC}{k_{B}T}(Z^{*}eE-\Omega \frac{d\sigma }{dx})$$

where J represents the flux of Cu ions, D is the diffusivity, C is the concentration, $k_{B}$ is Boltzmann’s constant, T is temperature, $Z^{*}$ is the effective charge number, e is the fundamental electronic charge, E=ρ×j is the electric field where ρ is the resistivity of Cu, j is the current density, Ω is the atomic volume, and $\frac{d\sigma }{dx}$ is the hydrostatic stress gradient along the line. 

Self-diffusion takes place within the boundaries of each interconnect domain as confined by the dielectric and metallic diffusion barriers used in the damascene technology. In this context, diffusion barriers block Cu self-diffusion across the interconnect boundaries, whereas self-diffusion dominantly takes place along Cu interfaces and grain boundaries [15]. Therefore, EM was modelled within interconnect domains isolated by the diffusion barriers, separately. Such domains may be composed of multiple segments with distinct electrical currents. 

Electromigration ageing can be subdivided into two main phases: (i) the pre-nucleation phase, where tensile stress builds up at the cathode end of interconnects until a critical stress required for void nucleation is reached and (ii) the post-nucleation phase, where the nucleated voids enlarge and evolve, increasing interconnect resistance. 

Considering the mass balance equation and using Equation (1), Korhonen derived a 1D partial differential equation (PDE) that describes the evolution of hydrostatic stress, σ, along an interconnect during EM [49]:

(2) $$\frac{\partial \sigma }{\partial t}=-\frac{d}{dx}\left[\frac{DBΩ}{k_{B}T}\left(\frac{Z^{*}eE}{Ω}-\frac{d\sigma }{dx}\right)\right]$$

where B is the effective elastic bulk modulus of the interconnect determined by considering the impact of confining layers and the dominant diffusion paths, i.e., the interfaces and the grain microstructure of the metal [24]. 

The physical input parameters for the EM model depend on interconnect dimensions, the type of dielectrics, and the barrier/liner/capping schemes [33]. The line width-dependent values of these parameters were previously reported in [33] and were employed in this study, as summarized in Table 1. As Equation (2) was solved numerically, the solution of the EM-induced stresses in multi-segment interconnects with different current densities was trivial and required assigning the relevant current densities to the domain of each segment, which were spatially discretized, while self-diffusion occurred across the segments and within the segments of an interconnect residing in a BEOL layer.

The fluxes at the two ends of the 1D electromigration domain are zero given the blockage by the diffusion barriers. Thus, by equating the total flux in Equation (1) to zero at the two ends, i.e., J=0, at x = 0 and x = L, the stress boundary condition for the solution of Equation (2) is derived as follows:

(3) $$\frac{d\sigma }{dx}=\frac{Z^{*}eE}{\Omega }$$

The initial stress was simplified and considered to be constant along the domain where technology-relevant values as in [33] were applied; see Table 1.

Once the EM-induced tensile stress calculated by the stress evolution PDE reached a critical stress, $\sigma_{crit}$, a void was considered to nucleate in the location, and a growth phase was initiated. Upon void nucleation, the stress at the void surface relaxes to zero to ensure the continuity of chemical potential energy [58]. This relaxation generates large stress gradients adjacent to the void surface, which drives diffusion from the void up the stress gradient, resulting in an increase in void volume. Korhonen suggested the use of a post-nucleation boundary condition at the cathode end of the interconnects, assuming that the void is exactly at the cathode end, to capture the flux of atoms from the void surface as follows [49]:

(4) $$J_{void}=\frac{D}{k_{B}T}(\frac{\sigma }{w})$$

where w is the width of the interconnect, assuming that the peak stress at the void relaxes to zero over a characteristic length equal to the width of the interconnect to estimate the stress gradient that drives atoms away from the void surface through fast diffusion paths. The application of this boundary condition for the solution of Equation (2) post-nucleation can capture the stress relaxation starting from the cathode void and its propagation to the rest of the interconnect, resulting in a shift of stress towards compression in the rest of the interconnect. In this case, the void volume at any time can be derived as follows [66]:

(5) $$V\left(t\right)=(\frac{A}{B})\int_{0}^{L}\left(\sigma \left(x,t\right)\right)dx$$

where A is the cross-sectional area, and L is length. In high-temperature accelerated tests on long interconnects, the void growth rate before volume saturation can be approximated as a function of the electron current density where the longitudinal drift velocity of an incubated void is as follows [15]:

(6) $$v_{d}=\frac{D}{k_{B}T}Z^{*}e\rho j$$

Once a void nucleated and started to grow, the resistance of the interconnect was calculated by assuming a growing void in Cu, while the interconnect cladding (i.e., combination of barrier and liner) still shunted the current around the void, as will be further described and formulated in Section 3.3.2.

### 3.3. Variability Propagation Modelling and Assumptions

EM-induced void nucleation and growth electrically manifests as an increase in interconnect resistance. In standard EM tests, interconnects are exposed to a constant DC current and once the resistance change exceeds a target value of 10%, the interconnect is considered to have failed. The variability of EM TTF stems from the variability of (i) time-to-nucleation and (ii) the resistive impact of voids depending on their location and morphology [67], as discussed in the following two sections. 

#### 3.3.1. Variability of Time-to-Nucleation at Single Interconnect Level

**Figure 3:**
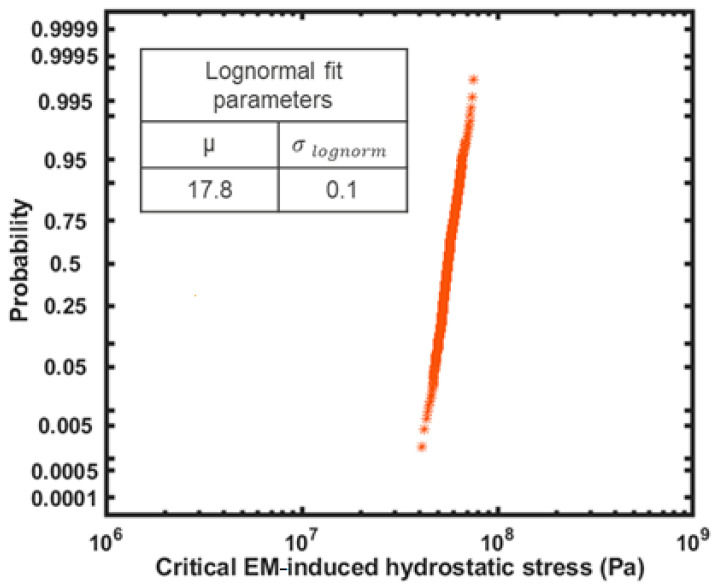
Distribution of critical electromigration-induced stress, $∆\sigma_{crit}$.

The intrinsic variability of time-to-nucleation is strongly correlated to the variability of the critical EM-induced stress to induce a void, i.e., $∆\sigma_{crit}$ [68]. Therefore, by deciphering the distribution of $∆\sigma_{crit}$, the employed Korhonen model is able to determine the variability of time-to-nucleation; see Figure 3. $∆\sigma_{crit}$ was assumed to follow a lognormal distribution [69] with strictly positive values. The median critical stress can be estimated based on the Blech characteristics of Cu interconnects, where, for interconnects of length L, there exists a critical current density j_c_, under which 50% of the tested population of interconnects show immunity to EM because the peak steady state stress does not exceed $∆\sigma_{crit}$ in half of the samples. The peak EM-induced stress along an interconnect is [68]:

(7) $$∆\sigma_{peak}=\frac{jLZ^{*}e\rho }{2\Omega }$$

By assigning j = j_c_, the median critical stress, $∆\sigma_{crit\_50\%}$, can be obtained as $∆\sigma_{peak}=∆\sigma_{crit\_50\%}$. The value of (jL)_c_ depends on many technology-dependent factors such as the adhesion of Cu at its interfaces, the mechanical properties and thickness of the cladding materials and dielectrics and interconnect dimensions [24]. For the interconnect considered in this study (width = 45 nm, height = 90 nm), a (jL)_c_ of 3300 A/cm was applicable [25], yielding a $∆\sigma_{crit\_50\%}$ of 56 MPa. Using a second point on the lognormal cumulative distribution function (CDF), the sigma of the lognormal distribution could be deciphered. This was estimated using our experimental insights, where, under a current density of 1.5 MA/cm^2^, immortality was never observed in interconnects longer than 25 μm. Using Equation (7), a peak EM-induced stress of 76 MPa was obtained, which was correlated to a high probability of 99%, to approximate a lognormal distribution for $∆\sigma_{crit}$, yielding a lognormal sigma of 0.1, as shown in Figure 3.

#### 3.3.2. Variability of Void’s Resistive Impact at Single Interconnect Level

Detailed physical modelling of the void’s interaction with current and stress fields, and thereby its impact on interconnect resistance, requires 2D and 3D void dynamics models such as in [52,57]. Such models are computationally expensive for modelling interconnect networks where many voids nucleate and simultaneously evolve within the network segments. Therefore, a compact model was devised to capture the resistance increase with void volume evolution and its variability in interconnect segments. The compact model was devised such that it could be conveniently calibrated from standard EM tests. Starting with the most simple case where the void behaves as a slice spanning across the Cu cross-section (henceforth referred to as slice-void) and increases its volume by drifting along the interconnect, the resistance of the interconnect will be as follows:

(8) $$R\left(t\right)=\frac{\rho_{MB}}{A_{MB}}\times l_{void}(t)+\frac{\rho_{cu}}{A_{cu}}\times (L-l_{void}(t))$$

where $l_{void}$ is the slice-void length, ρ is resistivity and MB denotes the metal barrier cladding surrounding the copper sidewalls and bottom interface. 

For a generic void morphology, which does not necessarily span across the interconnect, an equivalent slice-void length, $l_{void\_eqv}$, can be defined such that the resistive impact of the void would be equal to a slice-void with a length, $l_{void\_eqv}$, as follows:

(9) $$l_{void\_eq}\left(t\right)=(1/\xi )\times \frac{V(t)}{A_{cu}}$$

where V(t) is the void volume and $A_{cu}$ is the Cu cross-sectional area of the interconnect and ξ is a parameter which determines the severity of the void’s impact on electron flow, which for simplicity is assumed to be time-independent. Therefore, by substituting $l_{void\_eq}$ for $l_{void}$ in Equation (9), the resistance evolution of the interconnect is as follows:

(10) $$R\left(t\right)=(1/\xi )\times \frac{\rho_{MB}}{A_{MB}}\times \frac{V(t)}{A_{cu}}+\frac{\rho_{cu}}{A_{cu}}\times (L-(1/\xi )\times \frac{V(t)}{A_{cu}})$$

where ρ is resistivity and MB denotes the metal barrier. ξ relates to the void’s location and morphology, given that the resistive impacts of a slit void of a given volume which spans under a via or a void within a via are significantly more than a trench void of the same volume residing far from the via [70,71]. By introducing ξ, such differences in the resistive impact of voids are considered. The variability of a void’s resistive impact can be captured by deciphering the statistical distribution of ξ. In this context, the variability of time-to-failure, TTF, is a function of the variability of time-to-nucleation, T_nuc_, and time-to-growth, T_gr_, where:

(11) $$TTF=T_{nuc}+T_{gr}$$

**Figure 4:**
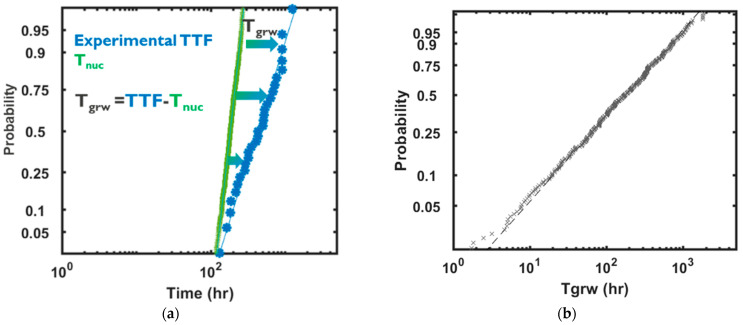
Calibration of T_gr_ for a given TTF distribution obtained from single interconnects. (**a**) TTF (blue) and T_nuc_ (green) distributions and (**b**) the obtained T_gr_ distribution.

As such, by having an experimental TTF distribution from standard EM tests on single interconnects, and by having the distribution of $T_{nuc}$ from the nucleation model, the distribution of $T_{gr}$ can be deciphered using Equation (11); see Figure 4.

**Figure 5:**
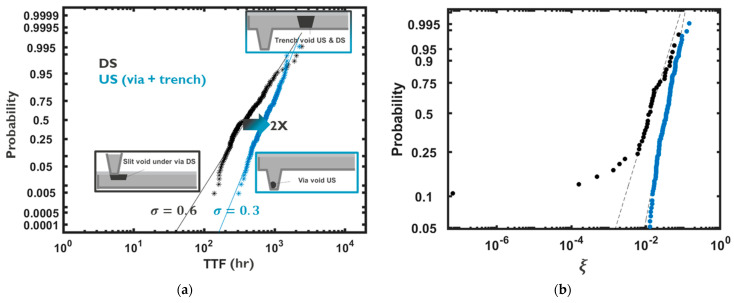
(**a**) TTF distributions based on upstream (US) and downstream (DS) EM tests on single interconnects and (**b**) the deciphered ξ distribution for the two cases.

Subsequently, using Equation (6), ξ can be calibrated from the distribution of $T_{gr}$ as follows:

(12) $$\xi =\frac{T_{gr}\times \frac{D_{eff}Z^{*}e\rho_{cu}j}{k_{B}T}\times \left[\frac{\rho_{mb}}{A_{mb}}-\frac{\rho_{Cu}}{A_{Cu}}\right]}{0.01\times \Lambda \times R_{0}}$$

where Λ is the failure criterion in percent based on resistence change, and $R_{0}$ is the initial interconnect resistence. Using Equation (10) and the T_gr_ distribution as shown in Figure 4, and considering Λ = 10%, the distribution of ξ was calibrated, as shown in Figure 5. The resistive impact of a near-via void is more pronounced than in trench voids. Especially in downstream EM tests, where electrons flow from a top via into a line underneath, it has been shown that the variability of TTF is larger than in upstream scenarios, where electrons flow from a via into a top line [70,71]. The increased variability of TTF stems from two distinct failure modes induced by (i) trench voids far from vias and (ii) under-via slit voids [70,71]. In Figure 5, the distribution of TTF for upstream and downstream scenarios for a given interconnect dimension, technology and current are shown. Fitting a lognormal distribution, a two-times-larger lognormal sigma is exhibited in the downstream scenario. Using the described approach, the distribution of ξ was obtained, showing that it follows a more monomodal distribution for the upstream case, whereas the distribution deviates from monomodality at lower tails of the distribution due to under-via slit voids, which results in an early increase in resistance and failure; see Figure 5. 

After addressing the EM variability at the single interconnect level by deciphering the distributions of $∆\sigma_{crit}$ and ξ, the impact of reduncy in interconnect networks was studied by employing the developed coupled electrical-EM modelling platform using the Monte Carlo approach. In each simulation instance, distinct values of $∆\sigma_{crit}$ and ξ from their respective distributions were allocated to interconnect segments within networks to obtain network-level TTF distirbutions.

In the results and discussion section, model predictions were initially corroborated by comparison to experimental data on double redundancy networks and subsequently the model was applied for the prediction of TTF distributions for a more complex PDN unit-cell (tile) with multiple redundant paths. 

## 4. Results and Discussion

### 4.1. Model Corroboration

**Figure 6:**
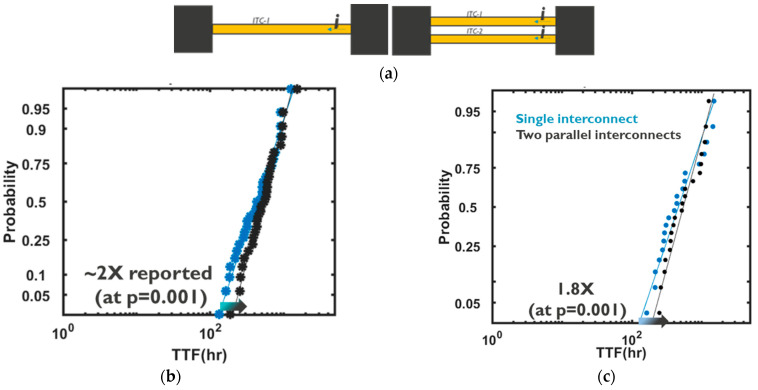
(**a**) The considered cases with a single interconnect and double interconnects. (**b**) TTF from Monte Carlo using experimental distributions. (**c**) Simulated probability plots of TTF distributions using the developed physics-based modelling framework. The continuous line is based on the lognormal fit to the data.

In order to corroborate the modelling framework for coupled electrical-EM analysis, the impact of redundancy was simulated and compared with experimental findings for a case with a single interconnect and a case with two interconnects in parallel; see Figure 6. The failure criterion of each interconnect was a 10% increase in resistance and the redundant case was considered to have failed when the last (toughest) interconnects failed. In addition, the input terminal current was scaled with the same factor as the number of redundant paths (i.e., increased by 2× for the case with two parallel interconnects); see Figure 6a. Therefore, initially, the interconnects were all conducting the same current, irrespective of the number of redundant paths. As shown in Figure 6b, a 2-fold increase in TTF for the case with double redundancy at a probability of *p* = 0.001 is expected, where the TTF distribution manifested a marked decrease in the lognormal sigma for the case with redundancy; see Figure 6b. This is because the TTF was defined based on the failure of the last surviving interconnect. From a statistical viewpoint, this can be understood, as by increasing the number of parallel redundant interconnects, the probability of having a more robust interconnect that lasts longer increases. Of note, this observation depends on the failure criterion. Conversely, if the failure criterion was the failure of the first (weakest) interconnect, the lognormal sigma would have decreased. Consistent with the experimental observations, the simulation framework predicted a 1.8-fold increase in the time-to-failure at *p* = 0.001 (Figure 6c), which consolidated the modelling framework’s predictions, quantitatively. In addition to the statistical reason behind the increase in TTF in the case with redundancy, a cascade of events was implicated in the observed enhancement. Upon void nucleation in the weakest interconnect and with its growth, the interconnect’s resistance increased and the current was redirected to the more robust interconnects. This, in turn, slowed down the ageing of the weakest link (i.e., the growth rate) and the tougher interconnect compensated by carrying larger current until it also endured void nucleation and growth. Obviously, if the input terminal current was not kept unchanged, i.e., if the currents scaled ½-fold for the double redundancy case, the lifetime extension would have been more significant. 

### 4.2. Application to PDN Unit-Cells

**Figure 7:**
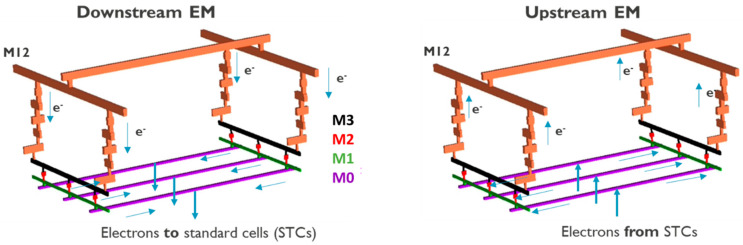
Schematic of the simulated network with the architecture of a PDN tile and its operation under upstream and downstream modes. The blue arrows indicate the direction of electron flow. Vertical and horizontal connections in each BEOL layer are referred to as vias and lines, respectively.

Following the corroboration of the simulation framework in the previous section, it was applied to simulate and predict EM ageing in a network based on the architecture of the PDN unit-cells in advanced CMOS technology nodes, where multiple redundant parallel paths exist; see Figure 7. In order to exclude Blech effect’s contribution that was previously shown to impact the TTF distributions of such PDN tiles [64], the tile dimensions and thereby the line lengths where considered to be relatively long (i.e., 100 µm), while the architectural and line segment length proportionalities were kept consistent. The BEOL stack consisted of 11 layers of interconnects where EM occurred in the lower-most layers of the stack, given their highest j/j_max_ ratio in the stack, i.e., in M0, M1, M2 and M3, which had a 45 nm linewidth and utilized the same dual damascene Cu interconnect technology. The EM voids occurred within the vias and lines if the EM-induced stress exceeded their assigned critical stress from the previously deciphered critical stress distribution in Section 3.3.1. Given the negligible length of the electron flow path within vias (i.e., 90 nm) compared to the length of lines (i.e., from 16 µm to 100 µm), and the direct correlation of stress with conduction length, i.e., $\sigma_{peak}\sim jL$ [49], the EM-induced stress within the via was approximated with the EM-induced stress in the line above the via (in a dual damascene scenario), as supported by our higher-dimensional EM simulations [59]. The M2 interconnect was considered to be a staple, i.e., the length of M2 was very short and functioned as a vertical connection between M1 and M3. Therefore, EM-induced stress in M2 was negligible and EM voiding was not thought to occur in the M2 staples. Three ideal current sources were connected to the network at the middle of M0, each supplying current to the three M0 lines at the bottom of the stack, analogous to the operation and configuration of standard cells in a CMOS chip. Upstream or downstream EM scenarios were investigated, where in the downstream case electrons flew from the highest metal levels to the lowest metal level and vice versa for the upstream case; see Figure 7. 

**Figure 8:**
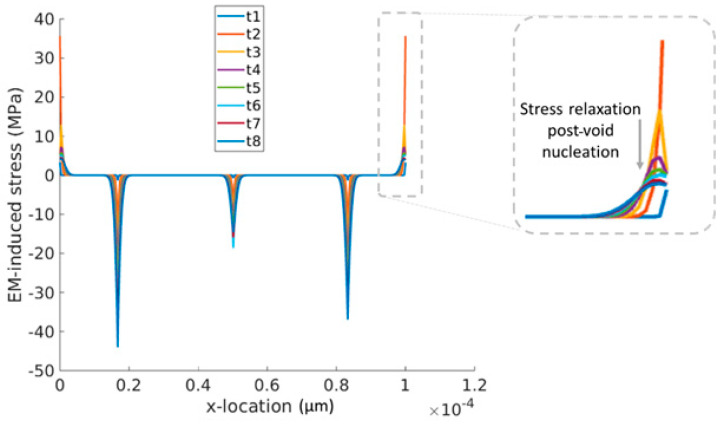
Transient stress distribution along M3 where void nucleation occurred at the two ends under the downstream operation mode. Colours depict different time instances spanning from pre- to post-void nucleation.

During the transient simulation, stress analysis was conducted for all interconnects at each time iteration of the explicit simulation from pre-nucleation to nucleation and post-nucleation, as shown in Figure 8. Prior to each simulation, a critical value of stress was assigned to every location within the interconnects in a Monte Carlo approach. As voids nucleated within the network and grew, the resistance of the voided segments increased and the initially symmetric current distribution was distorted, given the probabilistic nature of voids that occurred at different segments. As shown previously, the redundant paths compensated for voided segments by carrying larger currents [72,73]. Thereby, the asymmetric current distribution induced by the increase in resistance in the voided segments resulted in the asymmetric evolution of stress. In Figure 8, the M3 line with multiple segments is shown where, under downstream EM, its two ends (where the vertical totems land on M3) endure maximum tensile hydrostatic stresses. When the stresses exceeded the critical stress assigned to the specific location on the interconnects, void nucleation occurred, followed by a drastic relaxation of stress at the nucleation sites; see Figure 8. 

**Figure 9:**
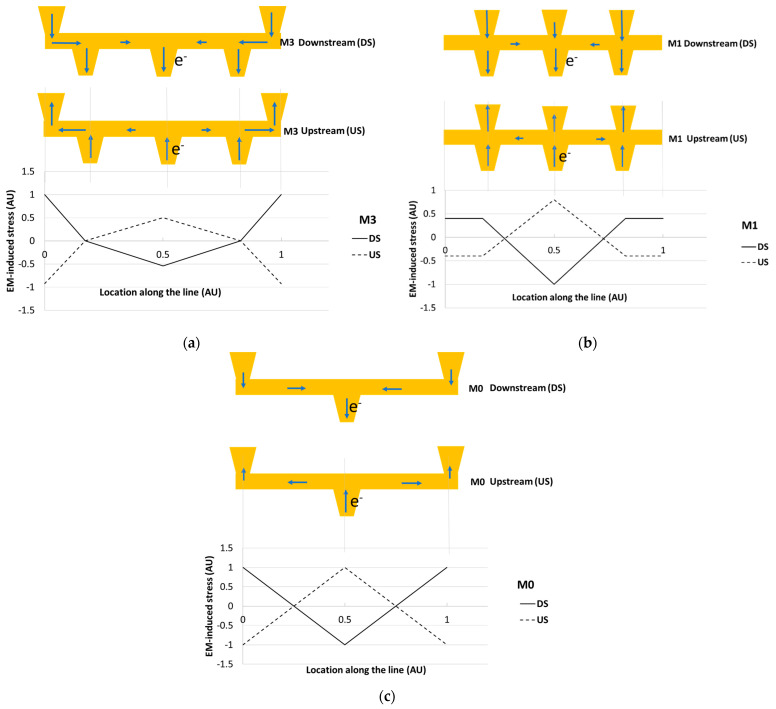
Stress distribution at steady state along (**a**) M3, (**b**) M1 and (**c**) M0 interconnects of the PDN tile shown in Figure 7. Stress normalized by the peak stress of each interconnect level. The schematics show the line–via configurations and the direction of electron flow under upstream and downstream operation.

**Figure 10:**
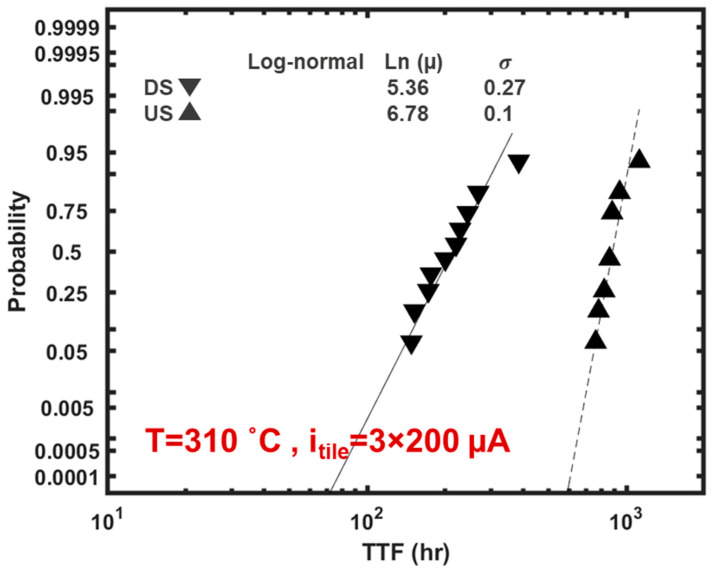
Simulated TTF distribution of the PDN tile shown in Figure 7, supplying the same total current under upstream (US) and downstream (DS) operation modes.

In addition, the stress distribution at steady state is of interest for tiles consisting of interconnects of very short lengths [48,64]. In such cases, the Blech effect will dominate in PDN tiles and results in the early immergence of a steady state where the net atomic flux along interconnects subsides to zero for interconnects operating below j_c_ [48,74]. In Figure 9, the stress distribution at steady state is shown. In M0, M1 and M3, the two ends endure the peak tensile stresses (positive hydrostatic stress) in the downstream operation, i.e., the V_ss_ grid. In contrast, their middle endures the highest tensile stress in upstream operation, i.e., the V_dd_ grid. Clearly, therefore, there is potentially a two times higher number of sites prone to EM voiding in the downstream case as compared to the upstream case, which contributes to higher EM vulnerability. In addition, for the specific grid configuration considered, in M3, the peak tensile stress has a magnitude that is two times larger in the downstream case than in the upstream case. The peak stress magnitude in M3 was 16.2 times larger than in M1; thereby, M3 was the interconnect level with highest EM-related events. This was mainly because the path from M3 to M0, passing vertically from the stacked vias at the top and the bottom of M1 and vertically through M1, constituted the least resistive path. Thereby, in the intact grid before EM voiding, the current magnitude along M1 is significantly lower than in M3 [72,73]. Given the observed stress distribution patterns, in the downstream scenario, the majority of voids were at the ends of M3 under V34 (i.e., the via connecting M3 to M4) and in the two ends of M0 where V01 (i.e., the via connecting M0 to M1) lands on M0. In contrast, in the US case the dominant failure mode was by voiding in the middle of M0 where the current source was attached and in M3 was either adjacent or in the three vias which connect M2 to M3 (i.e., V23). Clearly, therefore, V_SS_ grids are more vulnerable to EM compared to V_DD_ grids. The wider TTF distribution of single interconnects due to under-via voids in the downstream operation mode, as discussed in Section 3.3.2 and shown in Figure 5a, is a key cause of the relatively higher vulnerability of V_SS_ grids. Nevertheless, the model also identifies the heightened peak tensile stress and the increased number of vulnerable locations as exacerbating the vulnerability of V_SS_ grids. In this context, in Figure 10, the TTF distribution as determined by the model is shown for the downstream and upstream operation of the same unit-cell under the same operational conditions with electron flow direction as the only difference. The PDN unit-cell failure criterion considered was a 10% peak EM-induced voltage drop among the three supply points in the middle of M0 lines. PDN unit-cell TTFs followed a lognormal distribution and in both upstream and downstream cases the lognormal sigma of the grid unit-cells, $\sigma_{logn-tile}$, showed a marked decrease compared to the lognormal sigma of the constituent single interconnects, $\sigma_{logn}$, with a 50% and 66% decrease compared to single interconnects for downstream (V_ss_) and upstream (V_dd_), respectively. In addition, at the tile level, $\sigma_{logn-tile}$ was three times higher for downstream (V_ss_) compared to upstream (V_dd_), whilst by contrast this difference was only 2-fold at the single interconnect level. A 4.13-fold increase of t_50%_ was observed at the tile-level for the upstream compared to downstream operation, which was also more pronounced than in the single interconnect level where the difference was only 2-fold; see Figure 5a and Figure 10. Metal extrusion due to compressive stresses [21] was not considered as extrusions were not experimentally observed.

### 4.3. Impact on Electromigration Reliability Margins

As explained earlier, the standard approach for the evaluation of interconnect systems’ EM reliability has been by employing SEB, where the weakest link statistics are employed to predict the failure probability at a given time [43]. To this end, (i) the DC-equivalent design electrical current density, j_design_, in every interconnect needs to be determined by using EDA tools and (ii) the EM current density limits of single interconnects, referred to as j_max_, needs to be determined from single interconnect EM experiments, where j_max_ is determined from the TTF distribution of single interconnects as defined by the maximum current density that can be tolerated where a target failure probability, F_n_, (e.g., 100 PPM) at the expected lifetime (e.g., 10 years) can be met. Subsequently, the interconnect system’s failure probability at the target lifetime, $F_{sys}$, is calculated using the weakest link statistics as follows [10]:

(13) $$F_{sys}=1-\prod_{i=1}^{N}\left(1-F_{n}\right)=1-{\left(1-F_{n}\right)}^{N}$$

Assuming the lognormal distribution of TTF, N is as follows [10]:

(14) $$N=\sum_{i=1}^{K}\frac{1}{2F_{n}}\left(1+Erf(\frac{Z+\left(\frac{n}{\sigma_{logn}}\right)Ln\left[\frac{j_{design}}{j_{max}}\right]}{\sqrt{2}})\right)$$

where K is the total number of interconnects, n is Black’s equation’s current exponent factor, $\sigma_{logn}$ is the lognormal sigma of the TTF of tested interconnects and Z is the Z-score corresponding to F_n_ [10]. 

**Figure 11:**
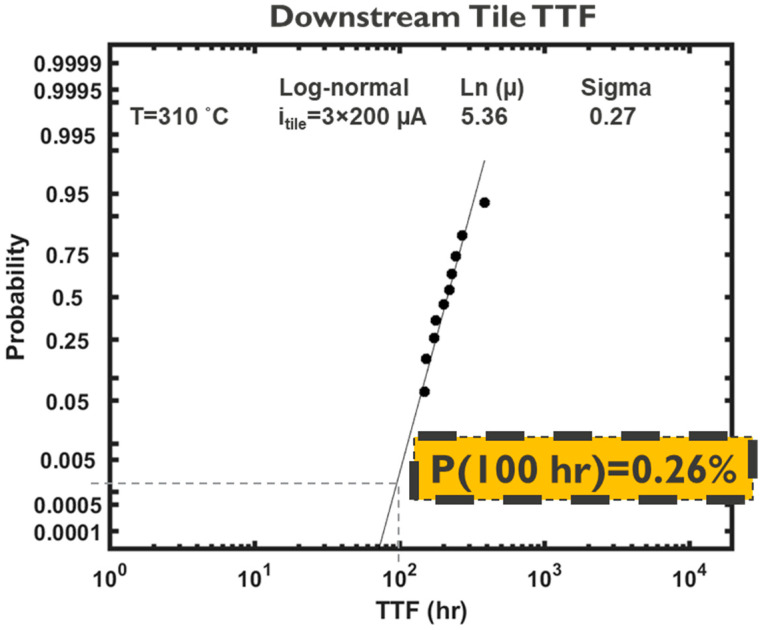
Cumulative distribution function (CDF) of the TTF of the simulated PDN tile shown in Figure 7, where the impact of redundancy is considered by employing the calibrated modelling framework. The data were fit with a lognormal function. For a target lifetime of 100 h, the probability of failure is 0.26%.

As previously discussed, an obvious limitation of this approach is that the failure of the first line is used to predict the failure of the interconnect system, which is an overly pessimistic assumption for grid-like networks such as PDNs. To evaluate the reliability margin neglected by SEB, the TTF distribution of the PDN tile described in the previous section was determined using the developed modelling platform; see Figure 11. A maximum voltage drop of 10% at the standard cell supply contacts (middle of M0) was considered as the failure criterion of the PDN tile. The TTF distribution was obtained under accelerated conditions with a temperature of 310 °C and a total PDN unit-cell/tile current of i_tile_ = 3 × 200 µA (i.e., 200 µA supplied to each M0 line), under downstream EM. As such, the failure probability predictions based on the obtained TTF distribution inherently capture the impact of redundancy in the PDN unit-cell. Interestingly, the lognormal sigma of the PDN tile’s TTF is 0.27 (Figure 11), as compared to 0.6 for the constituent interconnects in isolation (Figure 5a). This 55% reduction in the lognormal sigma is a consequence of redundancy and is consistent with the findings of the simple case shown in Section 3.1, as in highly redundant systems the TTF of the system is more strongly dictated by the TTF of the toughest link, rather than the weakest link.

A lognormal distribution was fit to the TTF distribution of the PDN tile and thereby the failure probability for an assumed target lifetime of 100 h was determined to be 0.26%; see Figure 11. Clearly, it is expected that a weakest link statistics approach results in a significantly more pessimistic system failure probability than 0.26%. For quantitative comparsion, the SEB was applied considering the same target lifetime of 100 h. In Table 2, the $j/j_{max}$ ratio and the statistical frequency for the PDN tile are summarized, where, consistent with the case shown in Figure 7, the total current of i_tile_ = 3 × 200 µA (i.e., 200 µA to each M0 line) was supplied to the PDN tile. The current densities in every interconnect segment of the PDN tile were obtained using the circuit solver. The current density limit, j_max_, of the single interconnects within the PDN tile under downstream EM was determined as follows [10]:

(15) $$j_{max}=j_{stress}{\left(\frac{{TTF}_{50\%,stress}}{t_{target}}\right)}^{\left(\frac{1}{n}\right)}e^{\left[\frac{Z\sigma_{logn}}{n}+\frac{E_{a}}{nk}(\frac{1}{T_{use}}-\frac{1}{T_{stress}})\right]}$$

where $j_{max}$ was obtained to be 1.1 MA/cm^2^ considering the downstream single interconnect test parameters: $\sigma_{logn}=0.6$, n = 1.5, activation energy E_a_ = 1.15 eV, $T_{stress}=T_{use}=310\;{}^{\circ}C$, ${TTF}_{50\%,stress}=400\;h$, $j_{stress}=1.5\;MA/{cm}^{2}$, the target lifetime $t_{target}=100\;h$ and Z = −3.1, which corresponded to F_n_ = 0.001. 

**Figure 12:**
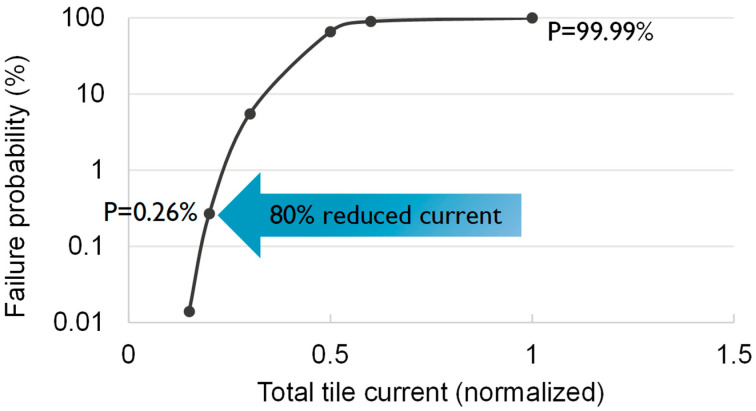
The impact of current on failure probability prediction by standard SEB (ignoring redundancy) for the PDN tile shown in Figure 7.

By employing the data summarized in Table 2 in equation 14, N = 10,025 was obtained. Thereby, using Equation (13), a failure probability of 99.99% is derived for the PDN based on SEB as clearly many interconnects have a current density higher than the limit; see Table 2. Of note, this failure probability is independent of the target single interconnect F_n_. For instance, choosing F_n_ = 0.1 corresponds to Z = −1.28 and thereby a less conservative $j_{max}$ of 2.26 MA/cm^2^ instead of 1.1 MA/cm^2^. This thereby leads to N = 100.9, which yields the same failure probability of 99.99%. Thus, the failure probability predictions by the standard SEB were starkly more pessimistic than the predictions considering redundancy, i.e., 99.99% vs. 0.26%, respectively. The practical implications of these paradigms can be more tangibly comprehended by considering that, in order to reduce the failure probability predicted by the standard SEB to a value of 0.26%, the input tile current had to be reduced by 80%, i.e., i_tile_ = 3 × 40 µA, instead of i_tile_ = 3 × 200 µA; see Figure 12. 

**Figure 13:**
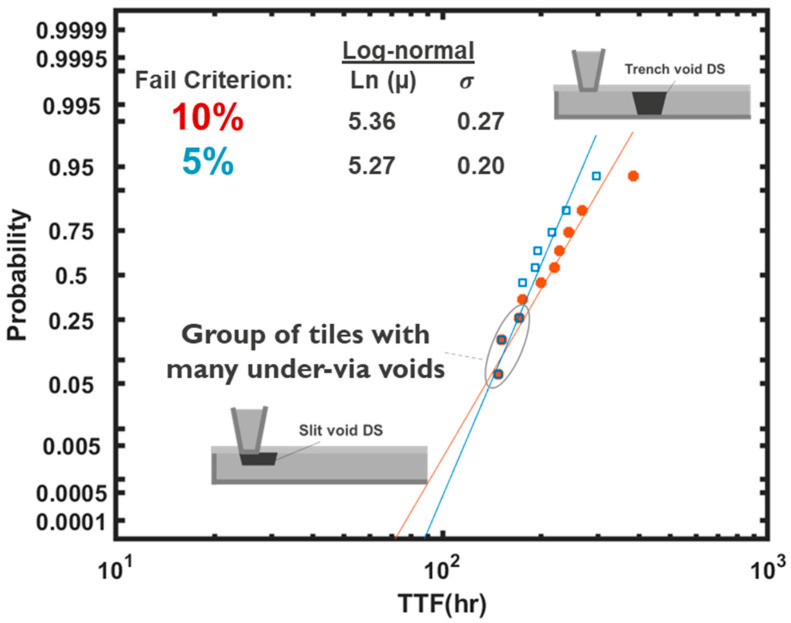
The impact of the failure criterion on PDN tile’s TTF distributions. The tile failure criterion is expressed as the peak EM-induced voltage shift in percent at the three current supply points in the middle of M0 lines, as shown in Figure 7.

In this analysis, a 10% peak EM-induced voltage drop at the PDN-tile level was used as the failure criterion. One might initially assume that adopting a more conservative voltage drop criterion, such as 5%, would increase the failure probability over a given lifetime, thereby reducing the identified reliability margin compared to the standard single interconnect-based approach. However, simulation results based on both 5% and 10% failure criteria in the downstream operation mode reveal that the TTF at the lower end of its cumulative distribution remains unchanged when the failure criterion is reduced to 5%; see Figure 13. This is attributed to the fact that the lower end of the TTF distribution is dominated by under-via slit voids, which lead to early catastrophic open failures upon nucleation. On the other hand, the upper end of the TTF distributions is primarily influenced by trench voids, which cause a gradual increase in the interconnect’s resistance as they drift along the trench. Therefore, a more conservative failure criterion only shortens the TTFs at the upper end of the distribution, while the lower end remains unaffected; see Figure 13. When fitting the TTF with a monomodal lognormal distribution, there is a 25% decrease in the lognormal sigma when the tile-level failure criterion is reduced from 10% to 5% of the EM-induced voltage drop, whereas a bimodal statistical fit would overlap at the lower tail region for 10% and 5% failure criteria.

Clearly, ignoring the identified reliability margins provided by grid redundancy imposes significant limitations on P&R resulting in an inevitably larger PDN real estate. It will also adversely impact chip performance and power consumption. In this context, the recently proposed approach [64] based on the characterization of the EM limits of PDN tiles as repeating unit-cells of PDNs instead of individual interconnects enables more realistic EM compliance evaluations of large VLSI PDNs. As such, by considering PDN tiles as the fundamental EM elements/links in SEB, the impact of grid redundancy would be inherently captured as redundancy is implicated in the PDN unit-cell current limits, i_tile_max_. To this end the current limit of a PDN unit-cell must be determined either experimentally by testing individual PDN unit-cells or by using efficient and predictive numerical models of PDN tiles, which are easily calibratable based on single interconnect tests. The modelling framework presented in this work is conducive to enabling the latter and determining the current limits of PDN unit-cells/tiles within the context of a tile-based SEB paradigm. The considered tile size is a crucial factor, given that if the tile-based EM characterization is conducted on tiles with the actual small dimensions of grid unit-cells, then short-length effects (SLE), such as the TTF saturation shown in [64], will manifest in characterizations and thus current limits. Alternatively, the tiles for characterization can be considered to have the architecture of the actual PDN unit-cells (thus, the same redundancy, connectivity and segment length proportionality) but with long interconnects (scaled) within each layer to minimize the influence of SLE. Obviously, the latter will result in relatively more conservative EM compliance checks compared to the former. However, from a design perspective, the latter would be the sensible approach as the power-rails in PDNs are mostly uninterrupted long multi-segment wires with multiple vias along their length. Thus, although the SLE assumption would be applicable for vertical connections (e.g., totems) across the BEOL stack layers, along the rails within a BEOL layer, SLE are not unequivocally certain. The latter approach would still provide significant reliability margins compared to the conventional single segment-based compliance checks, by considering grid redundancy.

## 5. Conclusions

This paper introduces a physics-based EM modelling framework that is complemented by experimental calibration. The framework is particularly suited for determining the current limits of unit-cells within nano-interconnect mesh networks, a critical step in enabling the recently proposed tile-based SEB paradigm [64] to assess the impact of nano-interconnect redundancy on chip reliability. The validity of the framework was confirmed through comparison with experimental data. For a representative PDN unit-cell/tile, the framework was employed to shed light on the EM reliability margin that would otherwise be overlooked by the single interconnect-based SEB. In this context, the variability of parameters involved in all stages of EM ageing, including the nucleation and growth phases, were addressed. Significant current margins were identified at the PDN unit-cell level, which exist due to unit-cell level redundancy and are disregarded by the conventional single interconnect-based methods. Furthermore, the study identified negative voltage power grids as more susceptible to EM. Vulnerable sites within a representative tile were examined based on mechanical stress distributions deciphered by the modelling framework. The study could pave the way for the adoption of tile-based SEB as a pragmatic approach for a more accurate prediction of chip reliability margins.

## Figures and Tables

**Table 1:** Interconnect geometrical and technological specifications and related physical input parameters for the electromigration model.

Parameter	Value	Description
$D_{a}$	Da(T = 310 °C) = 1.82 × 10^−20^ m^2^/s	Atomic diffusivity
ρ	49 Ω.nm	Resistivity
B	15 GPa	Interconnect effective bulk modulus
w	45 nm	Linewidth
h	90 nm	Line height
$Z^{*}$	3	Effective charge
e	1.60218 × 10^−19^ C	Electronics charge
Ω	1.182 × 10^−29^ m^3^	Atomic volume

**Table 2:** Breakdown of (jdesignjmax) ratios for all the single interconnects within the PDN tile shown in Figure 7 and the number of interconnects with the corresponding ratio shown as frequency. Both line and via values are listed.

jdesignjmax	0	0.58	0.63	0.94	1.2	1.89	3.05	3.63	4.58
Freq.	4	4	4	4	8	2	12	4	4

## Data Availability

The original contributions presented in the study are included in the article, further inquiries can be directed to the corresponding author.
